# Substrate-independent expression of key functional genes in *Cycloclasticus pugetii* strain PS-1 limits their use as markers for PAH biodegradation

**DOI:** 10.3389/fmicb.2023.1185619

**Published:** 2023-06-29

**Authors:** Anjela L. Vogel, Katharine J. Thompson, Daniel Straub, Constantin B. App, Tony Gutierrez, Frank E. Löffler, Sara Kleindienst

**Affiliations:** ^1^Department of Geosciences, Eberhard Karls University of Tübingen, Tübingen, Germany; ^2^Department of Environmental Microbiology, Institute for Sanitary Engineering, Water Quality and Solid Waste Management (ISWA), University of Stuttgart, Stuttgart, Germany; ^3^Quantitative Biology Center (QBiC), Eberhard Karls University of Tübingen, Tübingen, Germany; ^4^Cluster of Excellence: EXC 2124: Controlling Microbes to Fight Infection, Tübingen, Germany; ^5^School of Engineering and Physical Sciences, Heriot-Watt University, Edinburgh, United Kingdom; ^6^Center for Environmental Biotechnology, University of Tennessee, Knoxville, TN, United States; ^7^Department of Microbiology, University of Tennessee, Knoxville, TN, United States; ^8^Biosciences Division, Oak Ridge National Laboratory, Oak Ridge, TN, United States; ^9^Department of Civil and Environmental Engineering, University of Tennessee, Knoxville, TN, United States; ^10^Department of Biosystems Engineering and Soil Science, University of Tennessee, Knoxville, TN, United States

**Keywords:** PAH-degrading bacteria, process-specific marker genes, transcript-to-gene ratio, biodegradation, marine environment, aromatic ring-hydroxylating dioxygenases

## Abstract

Microbial degradation of petroleum hydrocarbons is a crucial process for the clean-up of oil-contaminated environments. *Cycloclasticus* spp. are well-known polycyclic aromatic hydrocarbon (PAH) degraders that possess PAH-degradation marker genes including *rhd3α*, *rhd2α*, and *pahE*. However, it remains unknown if the expression of these genes can serve as an indicator for active PAH degradation. Here, we determined transcript-to-gene (TtG) ratios with (reverse transcription) qPCR in cultures of *Cycloclasticus pugetii* strain PS-1 grown with naphthalene, phenanthrene, a mixture of these PAHs, or alternate substrates (i.e., no PAHs). Mean TtG ratios of 1.99 × 10^−2^, 1.80 × 10^−3^, and 3.20 × 10^−3^ for *rhd3α*, *rhd2α*, and *pahE*, respectively, were measured in the presence or absence of PAHs. The TtG values suggested that marker-gene expression is independent of PAH degradation. Measurement of TtG ratios in Arctic seawater microcosms amended with water-accommodated crude oil fractions, and incubated under *in situ* temperature conditions (i.e., 1.5°C), only detected *Cycloclasticus* spp. *rhd2α* genes and transcripts (mean TtG ratio of 4.15 × 10^−1^). The other marker genes—*rhd3α* and *pahE—*were not detected, suggesting that not all *Cycloclasticus* spp. carry these genes and a broader yet-to-be-identified repertoire of PAH-degradation genes exists. The results indicate that the expression of PAH marker genes may not correlate with PAH-degradation activity, and transcription data should be interpreted cautiously.

## Introduction

Polycyclic aromatic hydrocarbons (PAHs) are ubiquitous in the ocean, toxic for organisms, accumulate in biomass, and many are considered human carcinogens (Environmental Protection Agency, 1993; Landrum et al., 2003; Nikolaou et al., 2009; Stading et al., 2021; Zhang X. et al., 2021). Microbial biodegradation of PAHs is a key mitigation process (Genovese et al., 2014; Overholt et al., 2016; Gutierrez, 2019a). Since the 1970s, microbial degradation of crude oil components, like PAHs, is well known and bacteria, such as *Cycloclasticus* spp., were identified as relying on these toxic compounds to satisfy their energy and carbon demands (Dyksterhouse et al., 1995; Yakimov et al., 2007; Cui et al., 2019).

*Cycloclasticus* spp. are some of the best studied aerobic PAH degraders that utilize various PAHs, including naphthalene, phenanthrene, and pyrene, as sources of carbon and energy. They can also make use of certain fatty or amino acids (Geiselbrecht et al., 1998; Staley, 2010), are considered ubiquitous in the marine environment (Lozada et al., 2008; Cui et al., 2014; Rizzo et al., 2019), and recognized as early colonizers of the plastisphere due to their potential plastic degradation capabilities (Popovic et al., 2017; Denaro et al., 2020; Yakimov et al., 2022). Several studies reported correlations between *Cycloclasticus* spp. abundances and the presence of hydrocarbons, e.g., during oil spills (Gutierrez et al., 2013; Rivers et al., 2013; Kleindienst et al., 2016; Zhou et al., 2022). For example, Dong et al. (2015) reported relative abundances of *Cycloclasticus* sequences of 0.2 and 64.5% in pristine versus contaminated Arctic sediments, highlighting their key role in degrading petroleum hydrocarbons, particularly PAHs (Yakimov et al., 2007; Dombrowski et al., 2016; Sieradzki et al., 2021). However, the *in situ* rates of PAH degradation and the environmental factors influencing these rates remain largely unknown.

Quantifying PAH-degradation rates through canonical methods are challenging to conduct *in situ*. These methods include time-series hydrocarbon quantification via liquid chromatography or gas chromatography coupled to mass spectrometry or flame ionization detector (Wilkes, 2010) and standardized routine methods that can be used to calculate *in situ* PAH-degradation rates are largely lacking. Therefore, PAH-degradation rates are quantified in the laboratory and often determined as mean values over longer incubation times (Bacosa et al., 2021; Nolvak et al., 2021), which leads to a knowledge gap regarding predictive understanding of the environmental fate of PAHs. Thus, alternative methods are required to improve monitoring of microbial PAH-degradation activities in the environment.

The ratio of mRNA to DNA of functional genes—i.e., the so-called transcript-to-gene (TtG) ratio—can serve as a cultivation-independent measurement that potentially correlates with per-cell degradation rates and could, thus, provide an *in situ* method for PAH-degradation rate estimation (Baelum et al., 2008; Brow et al., 2013; Knapik et al., 2020; Tentori and Richardson, 2020; Bagi et al., 2022). The TtG ratio can identify real, per cell change (or lack thereof) in microbial activity (Baelum et al., 2008; Brow et al., 2013; Tentori and Richardson, 2020) as it corrects gene transcription against the potential overestimation of activity due to growth. Other studies have used volumes of culture medium or matrix, weight/concentration of protein, or cell counts as normalizing parameters (Baelum et al., 2008; Kazy et al., 2010; Kaya et al., 2019; Michalsen et al., 2021). However, using target gene abundances for normalization is advantageous because DNA and RNA can be extracted simultaneously and both can be analyzed in one assay, keeping biases due to different techniques to a minimum.

The TtG ratio can be determined by targeted methods, particularly quantitative PCR (qPCR) that is frequently used to assess the metabolic potential (DNA-based assays) or the transcriptomic response (RNA-based assays) of specific microorganisms or microbial communities to a biogeochemical perturbation, such as a hydrocarbon contamination event by targeting characteristic functional genes (McKew et al., 2007; Yergeau et al., 2009; Genovese et al., 2014; Knapik et al., 2020; Bagi et al., 2022; Liang et al., 2023). Further, as long as the TtG ratio is normalized by a quantitative measure (i.e., qPCR data of a housekeeping gene), even transcriptomes and meta-transcriptome data can potentially inform about degradation rates. Such an approach avoids “false positives” in transcription-based prediction of cellular activity (Zhang Y. et al., 2021). It remains, however, largely unknown if the TtG ratio is a useful proxy for PAH-biodegradation activity and, by extension, crude oil degradation.

Two characteristic enzyme classes involved in PAH degradation are aromatic ring-hydroxylating dioxygenases (RHDs) and PAH hydratase-aldolases (Martin et al., 2013; LeVieux et al., 2016; Lancaster et al., 2023; Yesankar et al., 2023). RHDs are ubiquitous in PAH degraders, catalyze the first rate-limiting step in the PAH-degradation pathway, and have a very conserved reaction site. The genes encoding for these enzymes are used as a functional marker for bacterial PAH degradation in multiple habitats such as soil, freshwater, and marine sediments (Iwai et al., 2011; Meynet et al., 2015; Liang et al., 2019b). Although the diversity of studied RHD genes in marine organisms is underrepresented, a detailed characterization, including substrate specificity of nine large (alpha) and nine small (beta) RDH subunits, was performed in a Pacific Ocean isolate, *Cycloclasticus* sp. strain P1 (Wang et al., 2018). The novel gene *rhd2alpha* as well as *rhd3alpha* (previously described as *phnA1* Kasai et al., 2003; McKew et al., 2007), which are large oxygenase subunits of RHDs catalyzing the first oxidation step in phenanthrene and naphthalene degradation, respectively, were identified as key enzymes for PAH degradation in *Cycloclasticus* sp. strain P1 (Wang et al., 2018).

Polycyclic aromatic hydrocarbon hydratase-aldolases catalyze a downstream step in aerobic PAH degradation, in which analogs of trans-o-hydroxybenzylidenepyruvate are transformed to aldehydes and pyruvate (Eaton, 2000). These enzymes are less substrate specific than RHDs, and the same hydratase-aldolase is involved in both naphthalene and phenanthrene degradation in *Cycloclasticus* sp. strain P1 (Wang et al., 2018). The *pahE* gene encodes a PAH hydratase-aldolase, which was proposed as a superior marker to identify PAH-degrading bacteria in comparison to RHDs because it is well conserved among PAH-degrading bacteria (Liang et al., 2019a). Assuming *pahE* is indeed involved in both naphthalene and phenanthrene degradation in *Cycloclasticus* spp., it would be an ideal candidate for a PAH-degradation functional marker gene (Liang et al., 2022). However, it remains to be determined if the single copy genes *rhd2α*, *rhd3α*, and *pahE* in *Cycloclasticus* spp. are suitable marker genes for PAH-degradation activity, if the transcription is substrate-dependent, and if the TtG ratios can potentially inform about activity (i.e., degradation rates). Thus, it is currently unclear if these functional genes and their transcripts can be used as a proxy of PAH degradation in environmental samples.

To deduce if the transcription of functional PAH marker genes in *Cycloclasticus* spp. can inform about PAH-degradation activity, we determined if (i) the TtG ratios of three functional PAH marker genes (i.e., *rhd2α*, *rhd3α*, and *pahE*) are correlated with the PAH-degradation rates observed in cultures of *Cycloclasticus pugetii* strain PS-1; (ii) the transcription of these marker genes in cultures of *Cycloclasticus pugetii* strain PS-1 is induced by the specific substrate (i.e., naphthalene or phenanthrene); and (iii) the genes and transcripts of these functional PAH marker genes can be found in crude oil-degrading seawater microcosms incubated under *in situ*-like conditions.

## Materials and methods

### *Cycloclasticus pugetii* strain PS-1 cultivation and PAH degradation

A freeze-dried culture of *Cycloclasticus pugetii* strain PS-1 (ATCC 51542), originally isolated from Puget Sound (Pacific Ocean) deep-sea sediments (Dyksterhouse et al., 1995), was obtained from the American culture collection (ATCC) and revived according to ATCC’s instructions. Cultures were maintained on nutrient-rich, artificial seawater medium (marine broth, Difco 2216, Sigma-Aldrich, United States), supplemented either with 200 mg L^−1^ naphthalene, phenanthrene, a mixture of both PAHs, or pyruvate as carbon and energy sources.

For the experimental setup, an inoculum of *Cycloclasticus pugetii* strain PS-1 was prepared by growing the strain with pyruvate as a carbon and energy source (0.003 mol L^−1^) in 30 mL of nutrient-rich, artificial seawater medium in serum vials (total volume 50 mL; acid rinsed, Milli-Q water rinsed, baked at 300°C for 8 h) crimped with PTFE-lined septa. From these 4-day old *Cycloclasticus pugetii* strain PS-1 pre-cultures, 300 mL of culture suspension was pooled (cell density 1.78 × 10^7^ ± 1.18 × 10^6^) and 1.5-mL volumes served as inocula for each of the biotic incubations for a total of 30 mL liquid in 50 mL glass serum bottles (acid rinsed, Milli-Q water rinsed, baked at 300°C for 8 h). For the PAH-containing batches, 50 μL of hydrocarbon substrates, dissolved in acetone, were added. In the cultures containing a single PAH, a final concentration of 200 mg L^−1^ naphthalene or phenanthrene was supplemented. In the cultures containing the PAH mixture, a final concentration of 100 mg L^−1^ each (both naphthalene and phenanthrene) was reached. The small amount of highly volatile acetone evaporated immediately after addition to the cultures and the PAHs were left as visible small crystals suspended in the medium. For these biotic incubations, six sacrificial, PAH-containing replicates were set up for each timepoint—one set of triplicates for PAH extraction and one set of triplicates for biological analysis. Additionally, sacrificial controls with pyruvate as a carbon equivalent to the carbon added in PAH-containing bottles (equal to pre-culture conditions) were set up in triplicates for biological analysis of DNA, transcripts, and cell numbers at timepoints of 0, 3, 6, 9, and 12 days. To evaluate potential abiotic degradation of the PAHs, non-inoculated duplicate controls were sacrificed at the first and the last day of the incubation period. All cultures were incubated in the dark on a rotary shaker at 125 rpm and at 18°C. Cell numbers, PAH concentrations, functional marker gene copies, and transcripts in the PAH-containing samples were quantified (see below) on days 0, 3, 4, 5, 6, 7, 8, 9, 10, 11, and 12.

### Quantification of hydrocarbons

We quantified naphthalene and phenanthrene concentrations over time with gas chromatography coupled to mass spectrometry (GC–MS). An Agilent 6890 N GC coupled to an Agilent 7973 inert MS, equipped with an Agilent 7683 B autosampler with a J + W Scientific DB-5MS (30 m length, 0.025 mm ID, and 0.25 μm film thickness) capillary column was used. The device was operated in a single ion mode with splitless injection and a helium flow rate of 0.8 mL min^−1^. An internal standard (D_8_-naphthalene and D_10_-phenanthrene) was added at a concentration of 20 mg L^−1^ to the respective PAH replicates and liquid–liquid extraction was performed immediately with 15 mL of cyclohexane of the whole, sacrificial vial. The samples were shaken for 30 min at 270 rpm, left undisturbed to separate the organic and the aqueous phases for a minimum of 3 days, before being diluted 1:1,000 with cyclohexane and analyzed by GC–MS. Degradation rates were calculated for each timepoint ( $r_{t}$ in mg L^−1^ day^−1^) using the change in concentration between the previous ( $C_{t-1}$ ) and the following ( $C_{t+1}$ ) timepoint: $r_{t}=\;\;|\frac{C_{t-1}-C_{t+1}}{\left(t-1\right)-\left(t+1\right)}\;|$ . This time-dependent degradation rate (in mg L^−1^ day^−1^) was used to monitor the change in degradation over the course of the experiment.

### Quantification of growth

Cell growth was quantified using two independent methods over the course of the experiments. One of these methods employed direct cell counting by taking 1 mL subsamples of culture suspension, fixing with 1% (v/v) paraformaldehyde, storing at 4°C before filtration (GTTP, 0.2 μm; Millipore), DAPI staining, and microscopic cell counts (Leica DM 5500 B epifluorescence microscope; Leica Microsystems, Wetzlar, Germany), as described previously (Rughöft et al., 2020). To compliment this method, functional marker genes were quantified as a measure of biomass, since *in silico* analysis using Geneious Prime (version 2019.1.1) and the NCBI data base (accessed in January 2019; Schoch et al., 2020) revealed that all investigated functional PAH-degrading genes were present as a single copy on the genome of *Cycloclasticus pugetii* strain PS-1 (Supplementary Table S1).

### DNA and RNA extraction, processing, and quality control

To follow DNA and RNA concentrations over time, between 15 and 30 mL of culture suspension (depending on cell density) were filtered on ice through 0.22 μm Sterivex filter cartridges (Merck Millipore, Darmstadt, Germany) and immediately frozen at −80°C for subsequent analysis. To account for DNA and/or RNA losses during extraction, luciferase RNA and DNA were added to the cartridges prior to extraction as an internal standard as described by Johnson et al. (2005). Briefly, 4.47 × 10^5^ copies μL^−1^ of luciferase DNA and 2.48 × 10^4^ copies μL^−1^ luciferase RNA were added to each Sterivex filter after thawing and prior to extraction. The recovered luciferase DNA and RNA were quantified by performing specific qPCR assays for each sample using the primers and protocol from Johnson et al. (2005). A DNA/RNA recovery efficiency was calculated for each sample to correct for losses during sample processing. For DNA and RNA extraction, the AllPrep RNA/DNA mini kit (Quiagen, Hilden, Germany) was used following the manufacturer’s instructions, but directly adding the extraction buffer to samples on the Sterivex filters. Extracted DNA was stored at −20°C and RNA at −80°C until further analysis.

To quantify transcripts, extracted RNA was purified by digesting the residual DNA with the TURBO DNA-free kit (Thermo Fisher Scientific Inc., Waltham, Massachusetts, United States) according to the manufacturer’s instructions. The purified RNA samples were transcribed to cDNA using SuperScript III reverse transcriptase (Thermo Fisher Scientific Inc., Waltham, Massachusetts, United States) and quantified by qPCR (in technical triplicates) together with the corresponding DNA samples.

### qPCR for functional genes

**Figure 1:**
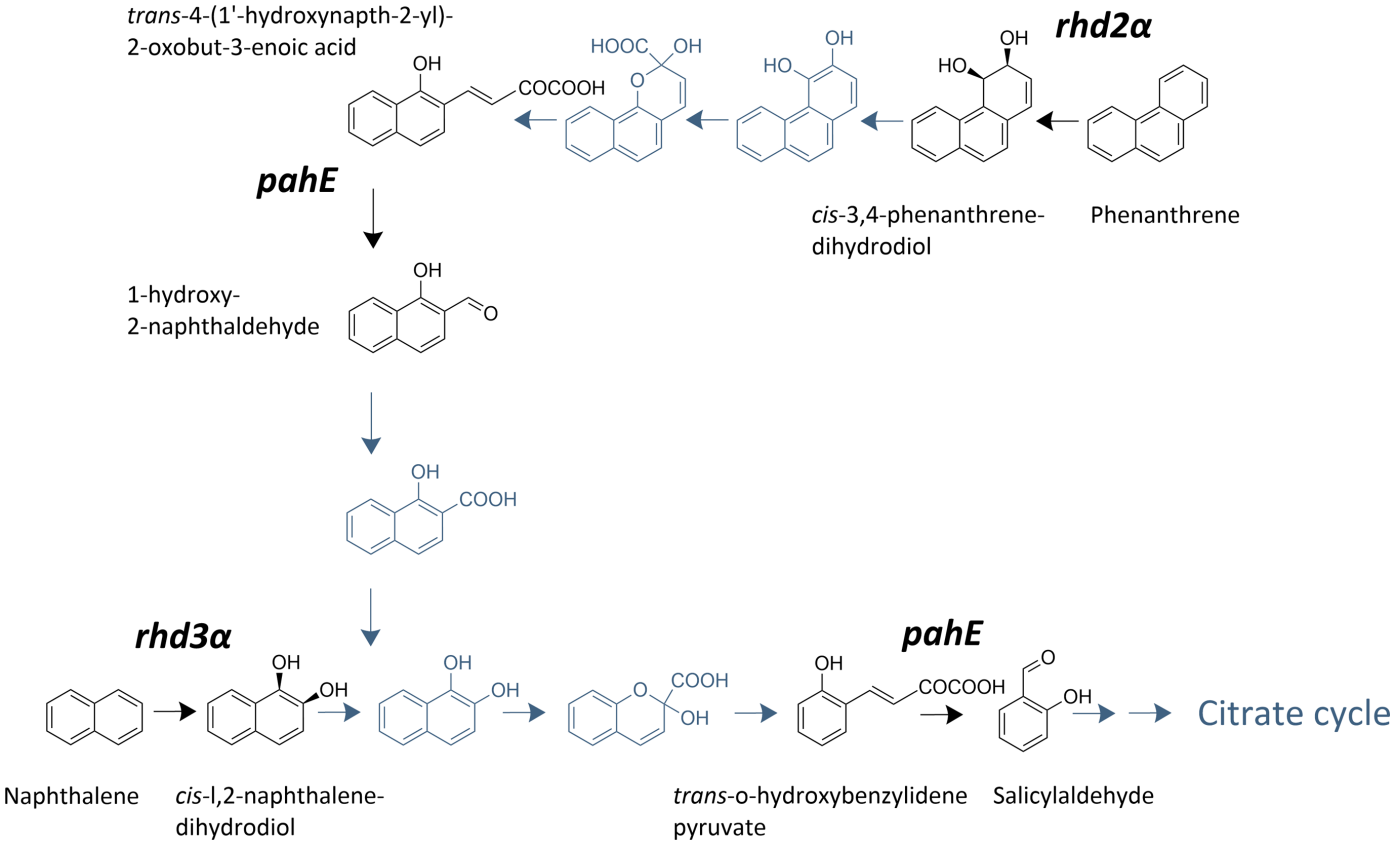
Polycyclic aromatic hydrocarbon (PAH)-degradation pathway used by *Cycloclasticus* spp. adapted from Wang et al. (2018). Steps carried out by enzymes encoded by the investigated genes are highlighted and labeled with the respective target gene.

Three functional marker genes, crucial for the PAH degradation in *Cycloclasticus* spp. (Wang et al., 2018; Liang et al., 2019a), were quantified (Figure 1): *rhd3α* specific for naphthalene degradation, *rhd2α* specific for phenanthrene degradation, and *pahE* involved in the degradation of both, naphthalene and phenanthrene.

Functional maker genes and their transcripts *rhd3α*, *rhd2α,* and *pahE* were quantified using qPCR. Primers for *rhd2α* and *pahE* were designed by identifying, collecting, and aligning all the nucleotide sequences of the respective genes from *Cycloclasticus* spp. available in the NCBI database (accessed in January 2019, Supplementary Table S1) and using the primer-design tool in Geneious Prime (version 2019.1.1). For several primer candidates, qPCR protocols were developed, and the best set of primers was selected for the following analysis [Tables 1, 2, following MIQE standards (Bustin et al., 2009; Supplementary Table S2)]. For *rhd3α*, we used published primers—Cyc372F/Cyc854R—and followed an established protocol (note that in the original publication *rhd3α* is referred to as *phnA1*; Dionisi et al., 2011). Quantification of DNA and RNA via qPCR was performed in technical triplicates for each of the biological replicates, and all replicates of DNA and RNA of the same sample were quantified on the same 96 well plate. Standards and negative controls were aliquots from the same batch and quantified on each plate to ensure reproducibility. Resulting gene and transcript abundances were corrected for extraction losses based on the recovery of the added luciferase DNA and RNA in the respective sample [i.e., for normalized DNA concentration of *rhd2α* (in copies mL^−1^) and respective recovery efficiency (in %); $\left[DNA_{rhd2\alpha }^{norm}\right]=\frac{\left[DNA_{rhd2\alpha }\right]*100}{recoveryefficiency}$ , Supplementary Figure S1]. Unitless TtG ratios were calculated by dividing transcript numbers (in copies mL^−1^) by gene abundances (in copies mL^−1^) to obtain a per cell measure for transcription (i.e., for *rhd2α*: $TtG_{rhd2\alpha }=\frac{\left[RNA_{rhd2\alpha }\right]}{\left[DNA_{rhd2\alpha }\right]}$ ). To confirm the amplified sequences were not false positives, Sanger sequencing was conducted on qPCR products of randomly selected samples for each functional gene and among the different experimental conditions.

**Table 1:** Primer characteristics for functional marker genes *rhd2α*, *rhd3α*, and *pahE*.

Target gene	Primer name	Primer sequence 5′– > 3’	T_m_ (°C)	GC content (%)	Hairpin temp. (°C)	Primer-dimer temp. (°C)	PCR prod.
*rhd2alpha* ^1,2^	rhd2α1126F	ACA CGA AGA GGA AAG CTG CA	59.9	50	x	1	199 bp
	rhd2α1305R	TTT TCT TGC CTG CAT AGC GC	59.8	50	42.9	x	
*rhd3alpha* ^2,3^	rhd3α 669FD	GGG TGG ACT AGC TGG AA	54.8	59	x	3.2	120 bp
	rhd3α781RD	TTC GCA TGA ATA GCG ATG G	55.9	47	59.1	11.2	
*pahE* ^1,4^	pahE 674F	TTG CTT GTA CAG GCC CTG AG	60	55	x	x	293 bp
	pahE 947R	CTC AGC CCA ACC TGT ACC AG	60	60	x	x	

^1^Primer design-this study, ^2^gene information (Wang et al., 2018), ^3^gene information (*rhd3α* = *phnA1*) and primer design from Dionisi et al. (2011), and ^4^gene information (Liang et al., 2019a,b).

**Table 2:** qPCR protocols for functional marker genes *rhd2α*, *rhd3α*, and *pahE*.

	*rhd2alpha*^1,2^	*rhd3alpha*^2,3^	*pahE*^1,4^
SYBR green	5 μL	5 μL	5 μL
Primer F (0.5 μM)	1 μL	1 μL	0.5 μL
Primer R (0.5 μM)	1 μL	1 μ	0.5 μL
H_2_O	2 μL	2 μL	3 μL
Nucleotide tmpl.	1 μL	1 μL	1 μL
Step 1	95°C, 5 min	95°C, 5 min	95°C, 5 min
	*1 cycle*	*1 cycle*	*1 cycle*
Steps 2–4	95°C, 20s	95°C, 20s	95°C, 20s
	62°C, 20s	62°C, 20s	58°C, 20s
		72°C, 20s	72°C, 20s
	*40 cycles*	*35 cycles*	*32 cycles*
Step 5	95°C, 1 min	95°C, 1 min	95°C, 1 min
	*1 cycle*	*1 cycle*	*1 cycle*
Step 6	62°C, 30s	62°C, 30s	62°C, 30s
	*1 cycle*	*1 cycle*	*1 cycle*
Step 7	62–95°C, 5 s steps	62–95°C, 5 s steps	62–95°C, 5 s steps
	*melting curve*	*melting curve*	*melting curve*

Volumes (10 μL in total) are given per reaction well. ^1^Protocol from this study, ^2^gene information (Wang et al., 2018), ^3^gene information (*rhd3α* = *phnA1*) and protocol adapted from Dionisi et al. (2011), and ^4^gene information (Liang et al., 2019a).

### Functional TtG ratios under simulated *in situ* conditions

To supplement our pure culture findings and test the transcription of the functional marker genes under environmentally relevant conditions, we analyzed selected Arctic microcosm samples (in biological triplicates). For the microcosms, water-accommodated fractions (WAF) of crude oil, prepared as described previously (Kleindienst et al., 2015), were used as the PAH source. The amendment of WAF to seawater-containing microcosms is a commonly used method since it allows the setup and analysis of homogeneous systems to simulate oil contamination in seawater. WAF contains a range of different total petroleum hydrocarbons (TPHs) with alkanes and PAHs. For instance, a previous study found that TPHs concentration in WAF-containing microcosms were around 300 μg L^−1^ with a naphthalene concentration of around 100 μg L^−1^ (Kleindienst et al., 2015). In the present study, for preparing the WAF, 150 mL of sweet light crude oil (Danish Underground Consortium) was added to 850 mL of pasteurized Arctic seawater and mixed for 48 h in the dark at 650 rpm, and then allowed to stand for the oil and water phases to separate. The aqueous phase, constituting the WAF, was separated and added to a total amount of 900 mL Arctic seawater at two different concentrations to allow the autochthonous microbial communities—which did not contain *Cycloclasticus* spp.—to respond to the PAHs in the WAF over a period of 32 days at 1.5°C in the dark. The treatments with low-pulsed WAF received 16 pulses of 80 μL WAF equally spaced over the incubation time of 32 days. The medium-concentrated WAF microcosms received an initial one-time dosage of 800 μL WAF at day 0. Dissolved organic carbon (DOC) was quantified in technical duplicates as an estimate of organic substrate in all microcosms. Therefore, 23 mL of sample were measured using a TOC analyzer (Elementar High TOC II, Germany) in the DOC mode with thermal oxidation at 680°C and CO_2_ quantification by an IR detector. To quantify gene and transcript abundances, 400 mL were filtered on ice through 0.22 μm Sterivex filter cartridges (Merck Millipore, Darmstadt, Germany) and immediately frozen at −80°C. Subsequently, DNA and RNA were extracted as described above using the AllPrep RNA/DNA mini kit (Qiagen, Hilden, Germany), the TURBO DNA-free kit (Thermo Fisher Scientific Inc., Waltham, Massachusetts, United States), and SuperScript III reverse transcriptase (Thermo Fisher Scientific Inc., Waltham, Massachusetts, United States). Taxonomy was determined using DNA- and RNA-based 16S rRNA (gene) amplicon sequencing as described previously (Caporaso et al., 2011). Absolute 16S rRNA gene copy numbers were quantified using a DNA-based 16S rRNA qPCR assay described previously (Lueder et al., 2022) and *Cycloclasticus* spp. absolute abundances were estimated by multiplying the DNA-based *Cycloclasticus* spp. relative abundances (based on amplicon sequencing) with the total 16S rRNA gene copy numbers (based on qPCR). For the present study, we selected those samples in which *Cycloclasticus* spp. could be detected, and we quantified the functional marker genes *rhd3α*, *rhd2α,* and *pahE* with the above-described qPCR protocols. Similar to the pure culture experiments, Sanger sequencing was used on qPCR amplicons of randomly selected DNA samples from each biological triplicate to confirm the identity of the amplicons and recognize false positives.

### Statistics

All statistical tests were performed in R version 4.2.3 (2023-03-15; R Core Team, 2023) and results thereof were documented in Supplementary Table S3. Significant difference was defined when *p* ≤ 0.05. When possible, ANOVA (Chambers et al., 2017) was performed with interaction term but repeated without in the case that the interaction was not significant. Following the ANOVA, *post hoc* test Tukey’s ‘Honest Significant Difference’ (Tukey’s HSD) was used (Tukey, 1949). Pearson correlation was performed with package Hmisc v5.0-1 (Harrell, 2023), and significance values were corrected for multiple testing to false discovery rate (fdr) with Benjamini-Hochberg method (Benjamini and Hochberg, 1995).

## Results

### Hydrocarbon degradation in axenic *Cycloclasticus pugetii* strain PS-1 cultures

**Figure 2:**
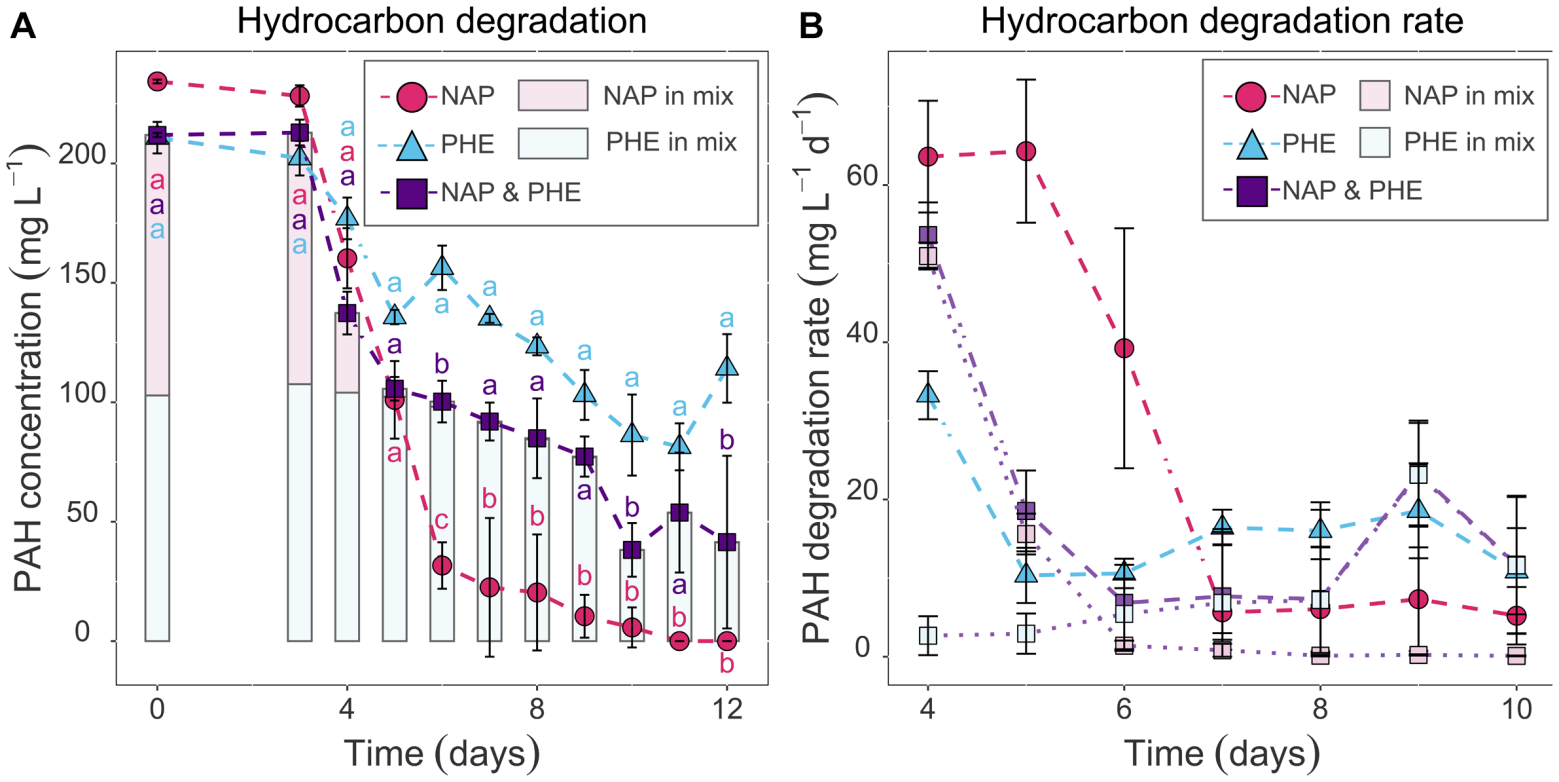
Hydrocarbon concentration quantified in sacrificial replicate serum bottles via GC–MS in mg L^−1^ **(A)** and the respective degradation rates in mg L^−1^ day^−1^ **(B)** over time. Comparison between naphthalene-and phenanthrene-only (pink circles and blue triangles, respectively) or mixed setups (purple squares represents total PAH concentration; light pink and light blue data show naphthalene and phenanthrene concentration in vials that received both PAHs). Error bars represent standard deviation. Letters indicate significant differences between PAH-amended treatments within a time point (ANOVA, *post hoc* test TukeyHSD, *p*_adj_ < 0.05), i.e., there is significant difference when letters do not intersect.

To determine if the TtG ratio of the investigated functional PAH marker genes can be used as a proxy for PAH-degradation rates and if the transcription of these genes is substrate specific, pure culture experiments were performed with *Cycloclasticus pugetii* strain PS-1, grown with naphthalene, phenanthrene, a mix of both PAHs, and in a PAH-free control. Pure cultures of *Cycloclasticus pugetii* strain PS-1, previously grown in PAH-free medium with pyruvate, degraded the provided PAHs after a short lag-phase of 3 days, while no growth or degradation was observed in abiotic controls (data not shown). In the single-compound incubations (PAH provided at 200 mg L^−1^), naphthalene was completely consumed (i.e., 86.5% between day 3 and 6; the remaining 13.5% until day 11) and naphthalene was consumed faster than phenanthrene, which was only degraded to approximately 50% after 12 days (Figure 2A). PAH concentrations differed significantly with time point (*p* < 2 × 10^−16^), provided substrate (*p* < 2 × 10^−16^), and the interaction thereof (*p* = 3.58 × 10^−15^), i.e., the PAH concentration changed significantly over time and dependent on the PAH addition (phenanthrene, naphthalene, or a combination of both). PAH concentrations within time points were not significantly different until day 5, but from day 6 on, phenanthrene addition retained consistently the highest PAH concentration, while naphthalene had the lowest. The combination of phenanthrene and naphthalene led to an intermediary PAH concentration that was significantly different to phenanthrene-only addition at days 6, 10, and 12, and to naphthalene-only at days 6–9 and 11 (Supplementary Table S3, Sheet A). The highest rates of naphthalene degradation of 64 mg L^−1^ day^−1^ occurred before day 5, after which the rates of degradation decreased until the naphthalene concentration dropped below 40 mg L^−1^ by day 6 (Figure 2B). Thereafter, naphthalene degradation activity remained at a constant rate of approx. 5 mg L^−1^ day^−1^ until the substrate was no longer detectable (Figure 2B). Similarly, the rate of phenanthrene degradation was higher in the beginning (33.3 mg L^−1^ day^−1^) but then remained relatively constant for the duration of the experiment (rates of 10–18 mg L^−1^ day^−1^; Figure 2B).

When provided with both naphthalene and phenanthrene (100 mg L^−1^ each), naphthalene was depleted rapidly to 2.1 mg L^−1^ by day 5, whereas phenanthrene degradation commenced around day 6 (Figure 2A); this was also shown in the degradation rates for the two PAHs in these incubations (Figure 2B). The degradation rate of naphthalene decreased from day 4 to 6 and was similar to that observed in the naphthalene-only experiments at an equal naphthalene concentration (51 and 1.4 mg L^−1^ day^−1^, respectively, Figure 2B). Conversely, the rates for phenanthrene degradation were low at day 4 and 5 (rates of 2.7 and 2.9 mg L^−1^ day^−1^) and progressively increased from day 6 onwards (Figure 2B). Following day 6, the residual naphthalene in the mixed-PAH experiment was degraded at rates of 0.1–1.4 mg L^−1^ day^−1^, with complete degradation achieved on day 10 (Figures 2A,B). Phenanthrene degradation activity increased slowly and reached a rate comparable to the phenanthrene-only experiment at 9 days (highest rate at day 9: 23.4 mg L^−1^ day^−1^; Figure 2B).

### Growth of *Cycloclasticus pugetii* strain PS-1 and its functional gene abundance and expression

**Figure 3:**
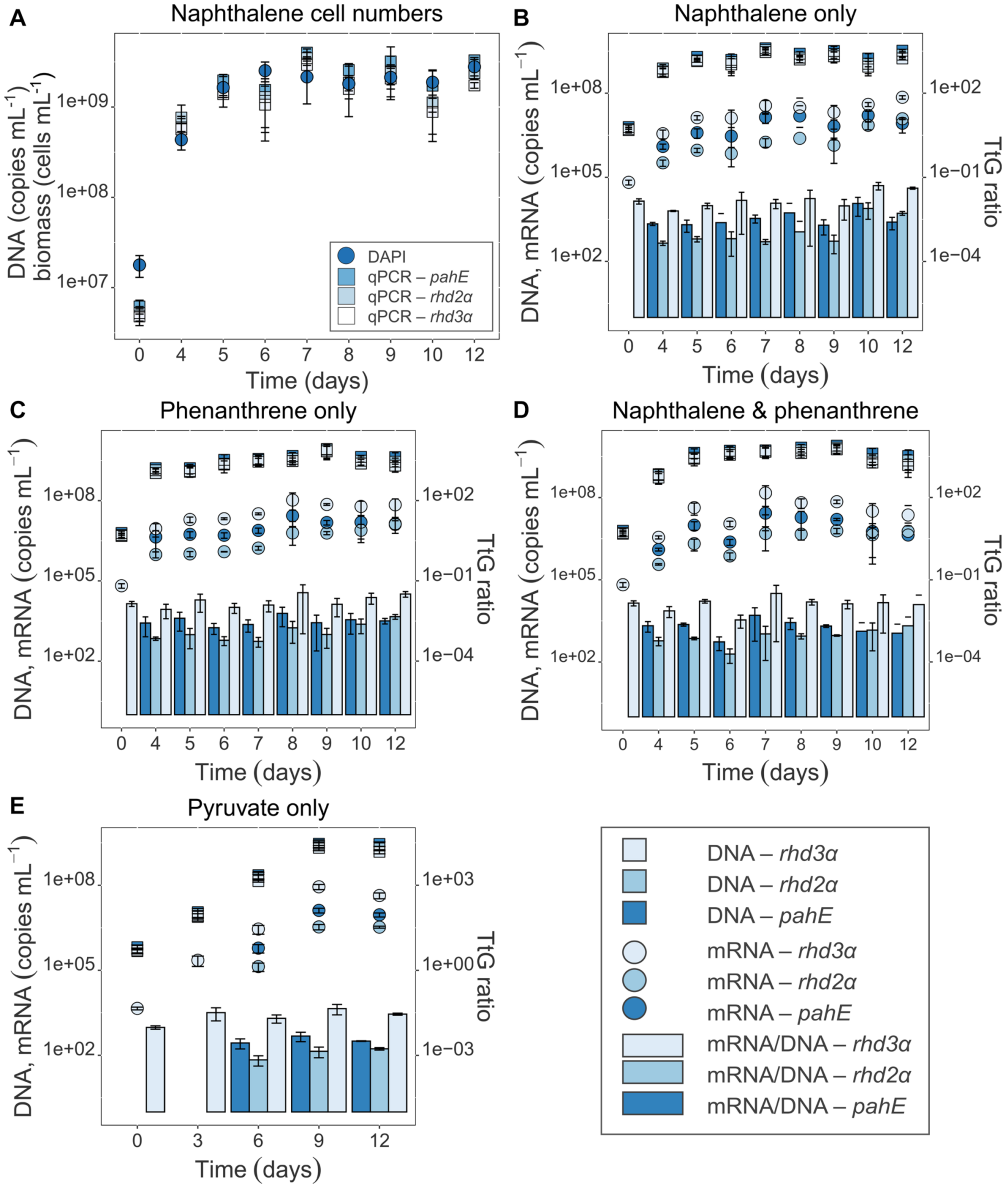
**(A)** Cell numbers over time for naphthalene-only experiments (all others are shown in the SI; Supplementary Figure S2). Gene-based copy numbers of *pahE*, *rhd2α*, and *rhd3α* (in copies mL^−1^) were quantified by qPCR and corrected for losses during DNA and RNA extraction by an internal standard (Johnson et al., 2005). DAPI-stained cells were counted and given in cells mL^−1^. **(B–E)** Target gene DNA and mRNA concentration (copies mL^−1^) of the functional genes *pahE*, *rhd2α*, and *rhd3α* are shown in the pure culture experiments amended with naphthalene only **(B)**, phenanthrene only **(C)**, naphthalene and phenanthrene **(D)**, and pyruvate only (control; **E**); (DNA—square, mRNA—circle); TtG ratios given as bar plots. Error bars indicate standard deviation; T_0_ mRNA concentration of *rhd2α* and *pahE* were below detection.

Cell counts using DAPI staining and fluorescence microscopy showed that *Cycloclasticus pugetii* strain PS-1, when supplemented with PAHs, reached stationary phase after 5–6 days [e.g., 4.74 × 10^9^ ± 1.39 × 10^8^ cells per mL were produced with mixed PAHs (Supplementary Figure S2)]. In the no-PAH controls supplemented with pyruvate, the cells reached stationary phase toward the end of the experiment between days 9 and 12 and 2.51 × 10^9^ ± 3.68 × 10^8^ cells per mL were produced (Supplementary Figure S2). The cell numbers correlated well with the copy numbers of the functional genes (Pearson’s correlation coefficients 0.698, 0.735, and 0.786 for *rhd3α*, *rhd2α*, and *pahE*, respectively; *p*_adj_ < 10^−15^, Supplementary Table S3, Sheet B, Supplementary Figure S3), as quantified via qPCR and corrected for the luciferase recovery efficiencies to adjust for extraction losses (naphthalene-only experiment shown as example, with a maximal number of 2.79 × 10^9^ ± 8.52 × 10^8^ cells per mL at day 12; Figure 3A). Luciferase qPCR results indicated that RNA and DNA recovery efficiencies both ranged between 3 and 30% (Supplementary Figure S1). While the total amount of extracted DNA was higher, RNA and DNA recovery efficiencies were similar, indicating an effective RNA extraction process. Transcripts of all functional genes could be quantified and were found to increase over time and bacterial growth (Figures 3B–E). The *rhd3α* transcripts were most abundant with a maximum of 10^7^ transcripts per mL determined between day 10 and 12, followed by *pahE* (maximum abundance of 10^6^ transcripts per mL between day 10 and 12), and *rhd2α* (maximum abundance of 10^6^ transcripts per mL between day 10 and 12, Figures 3B–E). Similar results were observed in all pure culture experiments regardless of the provided substrate (naphthalene, phenanthrene, or pyruvate). Cultures amended with pyruvate reached a maximum abundance of 10^5^ transcripts per mL for *rhd2α* between day 9 and 12 (Figure 3E). When normalizing the transcript copy numbers to the respective gene abundances, there was no significant change of the TtG ratios until day 9 (Figures 3B–E; Table 3), indicating per-cell expression of all investigated functional genes lacked a response to the initial PAH-degradation activity in all pure culture experiments. Although there was no initial change of the TtG ratio for all genes and treatments, the TtG ratio for *rhd3α* and *rhd2α* changed significantly over time (*p* = 5 × 10^−5^ and *p* = 10^−8^, respectively) when comparing each gene separately over all provided substrates at all common measured time points (*rhd3α*: 0, 6, 9, 12 days; *rhd2α* and *pahE*: 6, 9, 12 days; Supplementary Table S3, Sheet C). Specifically, the TtG ratio at day 12 for the naphthalene-only treatment, when substrate had been completely consumed and the bacteria were starving, was significantly higher compared to earlier TtG ratios of the same treatment (Supplementary Table S3, Sheet C). For pyruvate-only amended cultures less and different time points were measured compared to the other treatments (0, 3, 6, 9, 12 days vs. 0, 4, 5, 6, 7, 8, 9, 10, 12 days), therefore, to allow more comparisons, TtG ratios were additionally compared separately for each gene and each provided substrate (Supplementary Table S3, Sheet D). The TtG ratio of *rhd3α* and *rhd2α* was constant in cultures with a combination of naphthalene and phenanthrene but varied significantly over time for naphthalene or pyruvate only (*rhd3α*: *p* = 0.0001 and *p* = 0.0295 respectively; *rhd2α*: *p* = 0.0004 and *p* = 0.0422, respectively; Supplementary Table S3, Sheet D). Furthermore, the TtG ratio for *rhd2α*, but not *rhd3α*, was significantly different over time (*p* = 0.0002) for the phenanthrene amendment (Supplementary Table S3, Sheet D). In cultures with naphthalene-only amendment, the *rhd3α* TtG ratio was significantly higher at days 10 and 12 (TtG-ratio average of both days 0.046) compared to any other time point (average of other days 0.012). The *rhd2α* TtG ratio, however, was only significantly higher at day 10 compared to any other time point (average of day 10 compared to the other days 0.007 vs. 0.001) in naphthalene-only treatments (Supplementary Table S3, Sheet D). In pyruvate amended cultures, differences were only significant between two time points each (Supplementary Table S3, Sheet D). The TtG ratio of *rhd3α* was significantly higher at 9 days (TtG-ratio average 0.044) compared to day 0 (average 0.010) and the TtG ratio of *rhd2α* was significantly higher at 12 days (average 1.7*10^−3^) compared to 6 days (average 6.9*10^−4^). The *rhd2α* TtG ratio was higher after 12 days of incubation (average 0.004) in phenanthrene-amended samples compared to any other treatments (average of other treatments average 0.001; Supplementary Table S3, Sheet D).

**Table 3:** Difference in TtG ratios of functional genes and transcripts in Arctic microcosm (low-WAF and medium-WAF) and pure culture experiments.

Target gene	TtG ratio in arctic microcosms		TtG ratio in *Cycloclasticus pugetii* strain PS-1			
	Experimental condition:		Growth condition:			
	WAF_low_	WAF_med_	NAP	PHE	NAP and PHE	PYR
*rhd2α*	4.61 × 10^−1^	3.63 × 10^−1^	2.10 × 10^−3^	1.58 × 10^−3^	9.92 × 10^−4^	1.25 × 10^−3^
*rhd3α*	n.a.	n.a.	1.95 × 10^−2^	1.90 × 10^−2^	1.43 × 10^−2^	2.69 × 10^−2^
*pahE*	n.a.	n.a.	3.94 × 10^−3^	3.31 × 10^−3^	2.16 × 10^−3^	3.55 × 10^−3^

Mean ratios calculated over all timepoints in treatments containing naphthalene (NAP), phenanthrene (PHE), both PAHs (NAP and PHE), or pyruvate (PYR) as the hydrocarbon-free control. Background color highlights the order of magnitude of the TtG ratio (from lighter to darker blue background indicating lower to higher TtG ratios).

### TtG ratios from crude oil-degrading seawater microcosms incubated under *in situ*-like conditions

**Figure 4:**
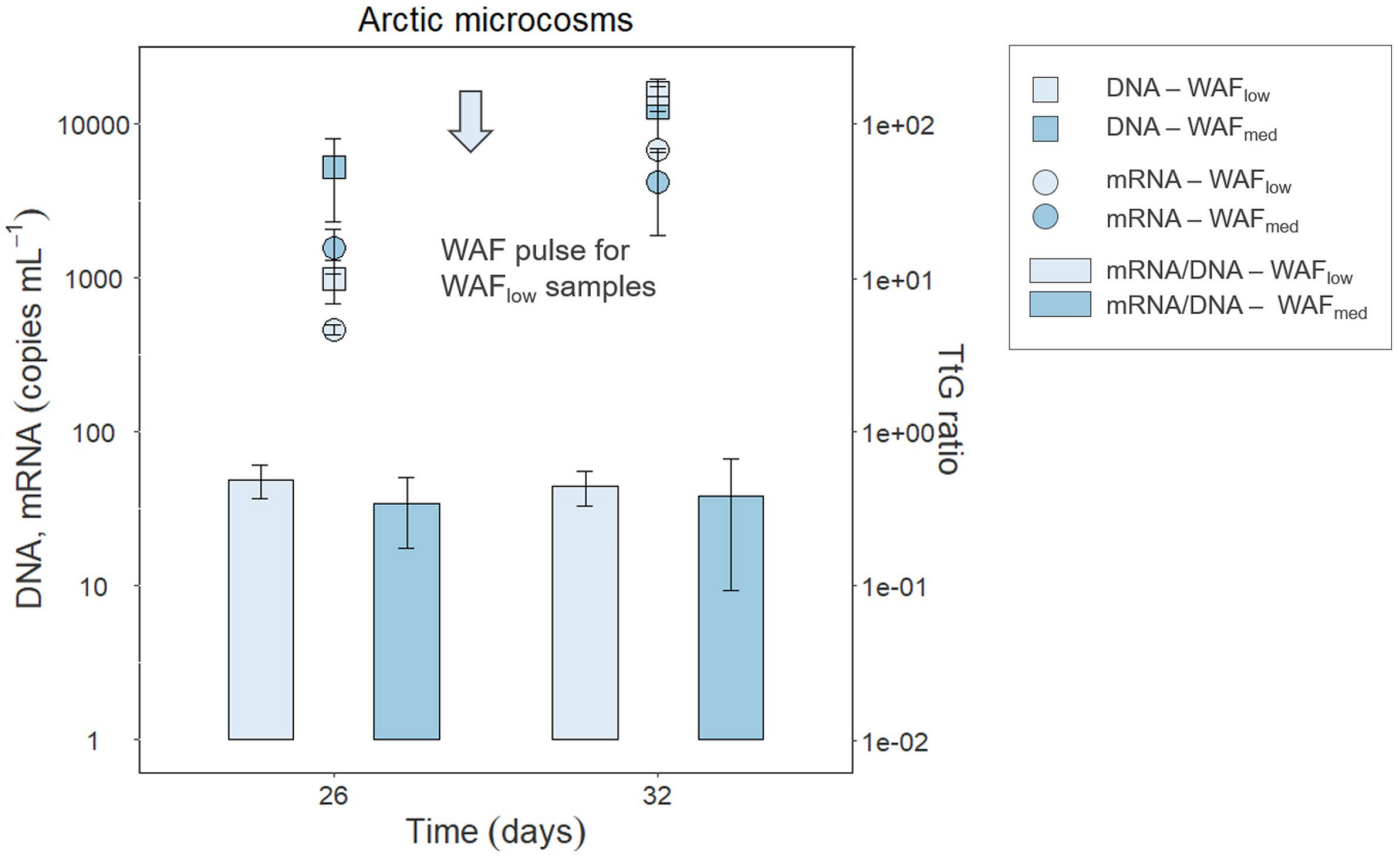
DNA and mRNA abundances (copies mL^−1^) of the functional gene *rhd2α* and its transcripts in Arctic microcosm samples with low (frequently pulsed) or medium hydrocarbon concentrations (DNA—squares, mRNA—circles); TtG ratios are shown as bar plots. Error bars indicate standard deviation.

To test if the genes and transcripts of the investigated functional PAH marker genes can be detected in crude oil-exposed microbial seawater communities, microcosms were set up using natural Arctic seawater and incubated under *in situ*-like conditions. To simulate crude oil contamination, the microcosms were amended with crude oil-based WAFs at two concentrations and the DOC was determined as a measure for available organic compounds. In the low WAF microcosms, DOC concentrations of 109.2 and 80.55 μM were determined at days 26 and 32, respectively. In the medium WAF microcosms, DOC concentrations of 90.63 and 90.72 μM were determined on days 26 and 32, respectively. Active *Cycloclasticus* spp. were identified in four Arctic seawater samples with absolute abundances of 1.62 × 10^3^ and 2.89 × 10^4^ copies mL^−1^ in low-WAF and 1.20 × 10^4^ and 3.85 × 10^4^ copies mL^−1^ in medium-WAF treatments, at 26 and 32 days, respectively. Although *rhd3α* and *pahE* could not be detected in samples taken from these microcosms, *rhd2α* genes and transcripts were detected and quantified in the investigated Arctic seawater microbial communities responding to WAF addition (Figure 4). Additionally, DNA and RNA were extracted from Arctic microcosm biotic controls, i.e., WAF-free treatments, and were tested with the developed qPCR assays. The target genes in these controls were below the detection limit, as expected, given that the abundance of *Cycloclasticus* spp. was also below the detection limit in these controls. WAF most likely contained a broader mixture of hydrocarbons (in addition to PAHs) and the increased transcript amount of *rhd2α* indicated phenanthrene degradation, given that *rhd2α* was shown to be essential for the initial step in this degradation pathway (Wang et al., 2018). The *rhd2α* qPCR results demonstrated that *Cycloclasticus* spp. were abundant and active as predicted from DNA-and RNA-based 16S rRNA (gene) sequencing microbial community results. Gene and transcript abundances of *rhd2α* significantly increased 4–5-fold between 26 and 32 days after WAF pulsing (*p* = 0.002 and *p* = 0.001, respectively). However, neither the amount of WAF (low vs. medium) nor the interaction between WAF and time (26 vs. 32 days) showed significant differences in gene or transcript abundance. In low-WAF microcosms, *rhd2α* gene and transcript abundances increased after WAF pulsing (between 26 and 32 days), leading to a constant TtG ratio of 4.87 × 10^−1^ ± 1.18 × 10^−1^ and 4.44 × 10^−1^ ± 1.12 × 10^−1^ at day 26 and 32, respectively (Figure 4). In medium-WAF microcosms, *rhd2α* gene and transcript abundances also increased, but to a lesser extent compared to the low-WAF microcosms, and yet a constant TtG ratio of 3.43 × 10^−1^ ± 1.66 × 10^−1^ and 3.84 × 10^−1^ ± 2.90 × 10^−1^ at day 26 and 32, respectively, was determined (Figure 4). The TtG ratio of *rhd2α* was therefore similar (mean of 4.61 × 10^−1^ ± 1.01 × 10^−1^ and 3.63 × 10^−1^ ± 2.12 × 10^−1^ for low-WAF and medium-WAF microcosms, respectively) across time points and experimental conditions, suggesting PAH-independent expression (Table 3) and neither time nor WAF concentration had any significant impact (Supplementary Table S3, Sheet E).

## Discussion

### Transcriptional responses as indicator for PAH degradation

Applying the TtG ratio to Arctic seawater microcosm experiments elucidated changes in activity (i.e., transcripts) due to the growth of *Cycloclasticus* spp. (Figure 4). Between day 26 and 32, the low-WAF microcosms received a last pulse (16 pulses in total) of crude oil-derived hydrocarbons that resulted in a higher *rhd2α* transcription, which could be interpreted as increasing PAH-degradation activity in response to the substrate pulse. When considering that the hydrocarbon pulse also led to growth of *Cycloclasticus* spp.—reflected in increasing *rhd2α* gene abundance—and using the growth-corrected TtG ratio, we found that the increase in *rhd2α* transcripts was due to an increase in cell numbers (i.e., growth), rather than increased transcription and degradation activity per cell.

Moreover, our results demonstrated a lack of transcriptional response to PAH-degradation activity for all investigated marker genes in *Cycloclasticus pugetii* strain PS-1. Further studies are required to assess if PAH-independent expression of the functional genes is followed by independent expression of the encoded enzymes and constant enzyme activities. Additionally, it remains unclear if there are other PAH-degradation genes in *Cycloclasticus* spp., or even in other important PAH-degrading taxa, that can be used to estimate PAH-degradation activity. Uncorrelated expression of PAH-degradation genes to PAH degradation highlights the importance of measuring and integrating additional (bio) geochemical data (i.e., quantifying PAH rates and the extent of degradation) in order to characterize the fate of PAHs.

### Influence of substrate types on PAH-degradation kinetics and marker gene expression

*Cycloclasticus pugetii* strain PS-1 preferred naphthalene over phenanthrene as a substrate, as indicated by higher degradation rates and preferential consumption in the mixed-PAH experiment (Figure 2). The higher degradation rate of naphthalene over phenanthrene could be explained by a difference in availability of the two substrates. One influencing factor could be the uptake of the PAHs into the cell. Current research suggests, however, that uptake of both naphthalene and phenanthrene into the cell is likely to occur via the same uptake systems (Yan and Wu, 2020). The respective dissolution kinetics, which are governed by the different physico-chemical properties of these substrates, on the other hand, can explain the difference in the availability of the two PAHs. Naphthalene is less hydrophobic, more soluble in the aqueous medium, and thus more bioavailable than phenanthrene (Yalkowsky et al., 1983; Volkering et al., 1992), which relates directly to the dissolution kinetics of these compounds, and in turn is the rate-limiting step for PAH degradation (Volkering et al., 1993; Johnsen et al., 2005). Our results demonstrated higher degradation rates for both naphthalene and phenanthrene in the beginning of the experiments when these substrates were each added alone, and for naphthalene in the mixed-PAH experiments, followed by a decrease in the degradation rate at constant high cell densities. These results support the degradation rate being limited by dissolution kinetics. This observation can further be explained since the dissolution of organic compounds—such as PAHs—also depends on the PAH particle size (Mulder et al., 2001), with smaller particles exhibiting faster dissolution rates that are, therefore, more easily accessible. Those smaller particles were potentially degraded in the beginning, supporting growth and activity of *Cycloclasticus pugetii* strain PS-1. At higher cell numbers and with aggregation of the remaining PAH particles and cells, mass transfer from pure PAH to the dissolved fraction became rate limiting, reflected in a decreasing PAH-degradation rate (Volkering et al., 1992; Mulder et al., 2001). To further untangle the limitations due to bioavailability, the quantification of the dissolved PAH fraction—i.e., by passive samplers in the cultures combined with modeling approaches—is necessary (Ribicic et al., 2018; Vogel et al., 2023).

In the mixed-PAH experiment, *Cycloclasticus pugetii* strain PS-1 exhibited signs of diauxic metabolism of naphthalene and phenanthrene. Prior research has reported diauxic utilization of mixed carbon sources (e.g., Gorke and Stulke, 2008; Wang et al., 2019). Some studies have reported that genes involved in the degradation of the secondary (less preferred) substrate are sometimes not expressed during the time when the primary (preferred) substrate is being utilized (i.e., carbon catabolite repression; Gorke and Stulke, 2008). Diauxic growth was observed in *Cycloclasticus pugetii* stain PS-1 (Figure 2), and while the initial steps of PAH degradation are unique, the remainder of the biochemical pathway is shared (Figure 1). However, diauxic growth likely did not occur due to carbon catabolite repression in *Cycloclasticus pugetii* strain PS-1 since both *rhd2α* and *rhd3α* were expressed independent of the available substrate (naphthalene, phenanthrene, and pyruvate). These findings further support that the higher bioavailability of naphthalene led to diauxic growth.

In all pure culture incubations, the TtG ratio of *rhd3α* was approximately one magnitude higher than that of *rhd2α*, which could further explain the preference by the strain for naphthalene over phenanthrene. But assuming that the higher TtG ratio of *rhd3α* over *rhd2α* is related to an increased cellular naphthalene-degradation activity does not seem likely for three reasons. First, the number of transcript copies per cell does not inform about enzyme cellular activity and further studies would need to show that a higher TtG ratio in *rhd3α* would correspond to a higher abundance of RHD3. To date, however, no data are available comparing different RHDs in a single organism and how transcription, enzyme activities, and resulting degradation rates are linked. Additionally, both *rhd2α* and *rhd3α* in *Cycloclasticus* sp. strain P1, as well as *phnA1* in *Cycloclasticus* sp. strain A5; encode RHDs that are not entirely specific for phenanthrene or naphthalene, as they can also transform other PAHs (Kasai et al., 2003; Wang et al., 2018). When expressed in *Escherichia coli*, the RHD3-genes enabled *E. coli* to degrade 83% of naphthalene and 21% of phenanthrene, while *E.coli* with RHD2-genes degraded 90% of phenanthrene and 21% naphthalene (Wang et al., 2018). This hints at a different affinity of dioxygenases for certain substrates, rather than specificity, meaning that the dioxygenases might display a high affinity for certain substrates but do not exclusively degrade them. It is also noteworthy that *rhd3α* and *rhd2α* are co-regulated in *Cycloclasticus* sp. strain P1 by the same CRP/FNR family regulator (Wang et al., 2021). Thus, preference of naphthalene over phenanthrene due to carbon catabolite repression linked to diauxic growth seems unlikely, and co-expression of *rhd3α* and *rhd2α* is not surprising.

Although co-regulation of the *rhd2α*, *rhd3α*, and even *pahE* might led to a lack of transcriptomic responses of *Cycloclasticus pugetii* strain PS-1 in all PAH-amended experiments regardless of the provided PAH substrate, the same outcome in the no-PAH controls cannot be explained. For *pahE*, there remains uncertainty if the encoded hydratase-aldolase is specific for PAH degradation. It cannot be ruled out that *pahE* is not involved in other metabolic pathways and was therefore expressed in PAH-free controls. RHDs, however, are well studied and several reports suggest that the expression of the corresponding genes is closely linked with PAH degradation (Cebron et al., 2008; Kweon et al., 2008; Paisse et al., 2012), and therefore the detection of those transcripts in PAH-free controls is unexpected. Inoculated cells in our study could have stored PAHs from the previous subculturing step, and as such these intracellularly stored PAHs might have been metabolized in the no-PAH controls resulting in the expression of these genes. However, potential intercellular storage of PAHs does not seem likely as the pre-cultures that served as inocula were grown on pyruvate, thus eliminating any possibility of transferring intracellularly stored PAHs. Additionally, the no-PAH controls were extracted and analyzed for PAHs, using GC–MS, which included the lysis of the cells to release any possible intracellular PAHs. From this, we did not detect any quantifiable PAHs in the no-PAH controls (data not shown). We thus conclude that *pahE*, *rhd2α*, and *rhd3α* expression is unrelated to PAH-degradation rates by *Cycloclasticus pugetii* strain PS-1 and that the expression is not influenced by the substrate.

We suspect that the PAH-independent expression of functional marker genes could be due to their co-regulation with a gene encoding a downstream enzyme that is essential to central metabolism. If the expression of this gene was regulated by the same regulator as the genes that are essential for the first steps of PAH degradation, PAH-independent expression of the whole pathway/functional PAH genes would not be surprising. As well as explaining substrate-independent expression of PAH-degradation genes, we speculate that this *Cycloclasticus* strain might also use an additional, yet unknown, set of genes for PAH degradation which might be expressed in concert to these three marker genes.

Considering *Cycloclasticus* spp. are described as hydrocarbonoclastic bacteria highly adapted to PAH degradation (Yakimov et al., 2007), the enzymes for PAH degradation are an essential part of the organisms’ lifestyle. This could lead to the corresponding genes, like the investigated functional marker genes, being PAH-independent or even constitutively expressed regardless of PAH availability. Indeed, the constitutive expression of hydrocarbon degradation genes was observed in other well-known hydrocarbon degrading bacteria (Cunliffe et al., 2006; Churchill et al., 2008). For example, *Mycobacterium* sp. strain CH-2 showed constitutive expression of genes encoding an alkane monooxygenase, whereas genes for PAH degradation were correlated with PAH availability and not constitutively expressed (Churchill et al., 2008). Since alkanes are presumably preferred substrates, the alkane monooxygenase might in this case be the key enzyme for the essential lifestyle of *Mycobacterium* sp. strain CH-2. The expression of *bphC* and *xylE* in *Sphingobium yanoikuyae* strain B1, which encode enzymes involved in naphthalene degradation, were also found to be constitutively expressed (Cunliffe et al., 2006). Although it is hard to discriminate the proportion of the increase in transcription due to cellular PAH-degradation activity from the proportion due to growing cell numbers of *Sphingobium yanoikuyae* strain B1, constitutive expression of the functional genes was especially prominent when the cells were grown in carbon-and nutrient-rich LB medium. This indicates that the essential marker genes for PAH degradation might be substrate-independent or constitutively expressed when cells are metabolically active and not starving. Starvation conditions are known to influence hydrocarbon degradation activity. For example, Rughöft et al. (2020) found that starvation conditions inhibited alkane degradation of *Marinobacter* sp. strain TT1, posing the question if PAH-independent expression of *pahE*, *rhd2α*, and *rhd3α* in *Cycloclasticus* spp. can also be observed under carbon-or nutrient-limited environmental conditions.

### Functional gene expression related to PAH degradation in an Arctic natural seawater community

The conclusion that transcription of the investigated functional genes alone is not a proxy for PAH-degrading activity was validated for a natural Arctic microbial community. We observed PAH-independent or possibly even constitutive expression of *rhd2α* in active (i.e., identified based on RNA) *Cycloclasticus* spp. from a natural community of Arctic seawater when amended with either low or medium concentrations of oil-derived hydrocarbons (Figure 4). PAH-independent expression of PAH-degradation genes in *Cycloclasticus* spp., therefore, was not only confirmed in carbon-rich laboratory incubations, but also suggested to occur in organisms of this genus in seawater from a natural marine environment.

We did not detect *rhd3α* and *pahE* genes or transcripts in *Cycloclasticus* spp. from the Arctic community, suggesting there is more variability in functional PAH-degradation genes in uncultivated *Cycloclasticus* spp. than expected from the similarity between the available sequenced genomes (e.g., genomes of *Cycloclasticus pugetii* strain PS-1 from this study and the well-studied *Cycloclasticus* sp. strain P1 share a pairwise nucleotide identity of 88.0%). Sanger sequencing of the amplified *rhd2α*-qPCR products from Arctic seawater samples showed 97% pairwise identity to the *rhd2α* sequence in *Cycloclasticus pugetii* strain PS-1. Although *rhd3α/phnA1* was previously used as marker gene for *Cycloclasticus* spp. in sediments (Lozada et al., 2008; Marcos et al., 2009; Dionisi et al., 2011), our findings suggest that *rhd2α* is potentially more ubiquitous in environmental *Cycloclasticus* spp. and could be a more robust marker gene to identify these organisms. What factors influence the functional variability of PAH degradation in *Cycloclasticus* spp. remains to be determined. Based on our results, comparing the isolate *Cycloclasticus pugetii* strain PS-1 to *Cycloclasticus* spp. from the Arctic microbial community, the two factors that might impact PAH-degradation rates are habitat (sediment vs. water column) and temperature (related to latitude; e.g., Gulf of Mexico vs. Arctic Ocean).

### Open questions on the application of TtG ratios

Collectively, the determination of TtG ratios could be a valuable tool in various microbiology disciplines in order to assess microbial activities linked to specific functional processes. In the omics-era, powerful methods have been developed and applied broadly and across diverse scientific disciplines, including metagenomics and meta-transcriptomics, to study differential gene expression patterns in response to environmental perturbations, such as an oil spill (e.g., Mason et al., 2012; Rivers et al., 2013; Tremblay et al., 2019). Further work could potentially apply the TtG ratio as a proxy of cellular activity on the transcriptomic or even metatranscriptomic level to investigate a process-specific set of target genes and thereby increase the validity and comparability of (meta-)transcriptomic studies. Normalization of (meta-)transcriptomic data by quantitative measures of a marker gene (i.e., 16S rRNA gene or a process-specific target gene) or absolute cell counts would be necessary in the process of TtG ratio calculation (Zhang Y. et al., 2021).

So far, there are still obstacles in using the TtG ratio as a proxy for PAH biodegradation under both laboratory-based experimentation and in field studies. Substrate-independent expression of the investigated functional PAH-degradation genes (i.e., *rhd2α*, *rhd3α*, and *pahE*) indicated that TtG ratios may not be applicable to predict PAH-degradation activity in *Cycloclasticus pugetii* PS-1 nor in closely related organisms. Additionally, determining the transcription of the functional marker genes on a single-cell basis would help to exclude the possibility that the apparent PAH-independent expression was an outcome due to averaging over both active and inactive cells in a culture. Moreover, there is a knowledge gap on the preference and regulation of RHDs for microorganisms, which have multiple gene copies or alternative RHDs genes in their genome. Additionally, RHDs are multi-component enzymes—there are three to four genes required to synthesize a functional RHD—and it remains to be tested if all of these genes are PAH-independently expressed. Future work should explore if there are additional PAH-degradation genes in *Cycloclasticus* spp. that are PAH-dependently expressed and whose expression correlates with decreasing substrate concentrations during degradation. This will help to determine if using different functional marker genes, enzyme activity assays, or (quantitative) proteomic approaches are more reliable proxies for biodegradation activity. Given that obligate hydrocarbonoclastic bacteria, like *Cycloclasticus* spp., have been found in pristine environments (Gutierrez, 2019b), the substrate-independent expression of PAH-degradation genes should be further investigated in order to avoid erroneously identified PAH-degradation activities.

## Conclusion

Overall, the TtG ratios of the investigated genes did not correlate with the PAH-degradation rates observed in pure cultures of *Cycloclasticus pugetii* strain PS-1. Furthermore, transcription of these marker genes in the pure cultures was not induced by a specific substrate—naphthalene, phenanthrene, or a no-PAH, carbon substrate alternative. In addition, we detected genes and transcripts of *rhd2α* in hydrocarbon-degrading Arctic seawater microbial communities during simulated oil contamination events under *in situ*-like conditions. The TtG ratio in the Arctic seawater was also independent of the PAH-degradation activity, similar to our findings from the pure culture experiments. Overall, PAH-independent, or possibly even constitutive, expression of the investigated genes suggested *rhd2α*, *rhd3α*, and *pahE* are not suitable target genes to predict PAH-degradation activity of *Cycloclasticus* spp. through RNA-based assays. Notably, not all the investigated genes were identified in the Arctic seawater microcosms, which highlights a current knowledge gap in the functional PAH-degrading gene diversity in environmental (i.e., Arctic) *Cycloclasticus* spp. In order to achieve quantification of PAH-degradation rates *in situ*, alternative target genes need to be identified whose transcriptional responses are tightly coupled to substrate availability. Ultimately, using the correlation of the TtG ratios of process-sensitive marker genes and PAH-degradation rates would allow the robust identification of contaminated areas and associated pollutant-biodegradation processes at high spatial and temporal resolution and it could further help to mitigate hydrocarbon pollution in marine environments.

## Data availability statement

The original contributions presented in the study are included in the article/Supplementary material (DataSheet ZIP); further inquiries can be directed to the corresponding author.

## Author contributions

AV, KT, and SK contributed to the study conception and design. The pure culture experiment was conducted by AV. The Arctic microcosm experiment was planned by CA and SK and conducted by CA and AV. AV, KT, TG, and SK were involved in data analysis and interpretation. Statistical analysis was performed by DS. The first draft of the manuscript was written by AV and all authors commented on previous versions of the manuscript. All authors contributed to the article and approved the submitted version.

## Funding

The authors acknowledge funding by the Emmy Noether Program of the German Research Foundation (Deutsche Forschungsgemeinschaft; DFG) granted to SK (grant number 326028733). KT is funded by the Institutional Strategy of the University of Tübingen (Deutsche Forschungsgemeinschaft; DFG/German Research Foundation, ZUK 63).

## Conflict of interest

The authors declare that the research was conducted in the absence of any commercial or financial relationships that could be construed as a potential conflict of interest.

## Publisher’s note

All claims expressed in this article are solely those of the authors and do not necessarily represent those of their affiliated organizations, or those of the publisher, the editors and the reviewers. Any product that may be evaluated in this article, or claim that may be made by its manufacturer, is not guaranteed or endorsed by the publisher.
