# Supplementary material for: Substrate-independent expression of key functional genes in Cycloclasticus pugetii strain PS-1 limits their use as markers for PAH biodegradation
Source: Front Microbiol. 2023 Jun 29;14:1185619. doi: 10.3389/fmicb.2023.1185619 (PMC10338962; doi:10.3389/fmicb.2023.1185619)
Supplement: Supplementary file 1 [file Data_Sheet_1.zip › Supplementary Table S1 and Supplementary Figures S1-S3.pdf]

Supplementing Information

TABLE S1 Gene sequence IDs of *rhd3α*, *rhd2α*, and *pahE* in *Cycloclasticus* spp. from the NCBI data base (accessed in January 2019) (Schoch et al. 2020) used for primer design. Pairwise nucleotide comparisons of target-gene sequences in the database to the respective gene sequence in *Cycloclasticus pugetii* strain PS-1 are also given.

***Cycloclasticus* spp. genome**	**Functional gene locus tag**	**Functional gene protein ID**	**Target gene**	**Pairwise identity**
C. pugetii strain PS-1	CYCPU_RS0111470	WP_016391028.1	*rhd3α*	100%
C. pugetii strain PY97N	CPC19_RS01670	WP_016391028.1	*rhd3α*	99.9%
Cycloclasticus sp. strain P1	Q91_RS11240	WP_015007028.1	*rhd3α*	99.7%
Cycloclasticus sp. strain TK8	SAMN05519226_0006	SHJ67137.1	*rhd3α*	98.3%
C. zancales strain 78-ME	CYCME_RS12395	WP_015007028.1	*rhd3α*	99.8%
C. pugetii strain PS-1	CYCPU_RS0111560	WP_015007046.1	*rhd2α*	100%
C. pugetii strain PY97N	CPC19_RS01580	WP_016390233.1	*rhd2α*	99.9%
Cycloclasticus sp. strain P1	Q91_RS11330	WP_016390233.1	*rhd2α*	98.3%
Cycloclasticus sp. strain TK8	SAMN05519226_1786	SHJ26042.1	*rhd2α*	98.3%
C. zancales strain 78-ME	CYCME_RS12485	WP_020933226.1	*rhd2α*	98.7%
C. pugetii strain PS-1	CYCPU_RS0105800	WP_015005964.1	*pahE*	100%
C. pugetii strain PY97N	CPC19_RS06820	WP_016390649.1	*pahE*	99.3%
Cycloclasticus sp. strain P1	Q91_RS05875	WP_015005964.1	*pahE*	99.6%
Cycloclasticus sp. strain TK8	SAMN05519226_2075	SHJ43395.1	*pahE*	99.1%
C. zancales strain 78-ME	CYCME_RS06220	WP_015005964.1	*pahE*	99.5%

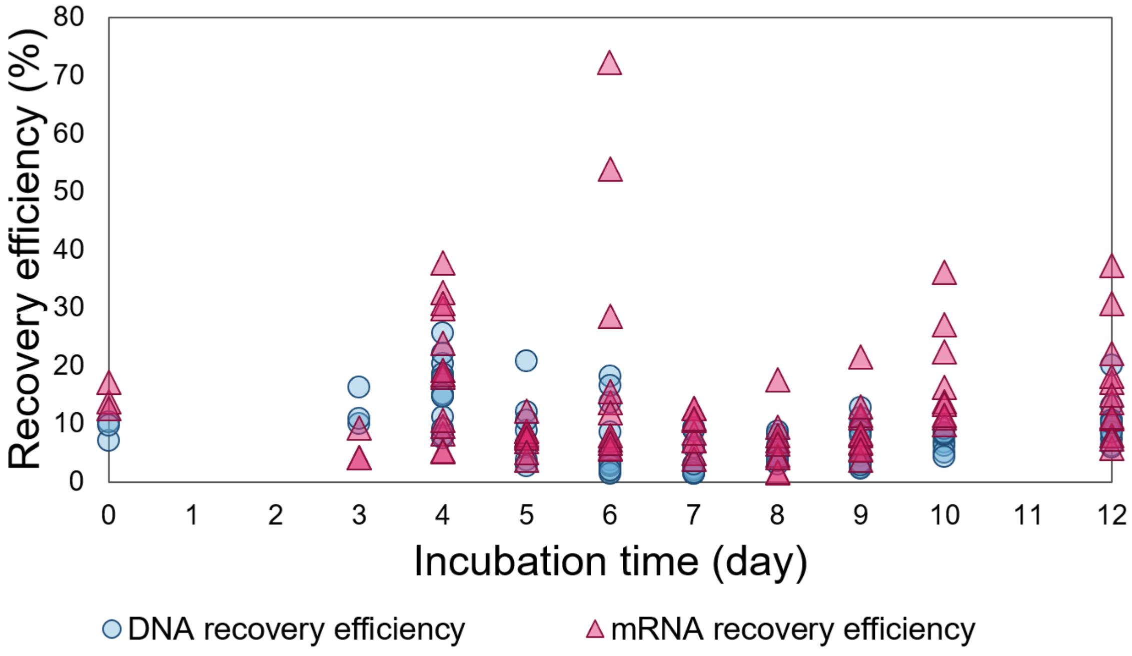

FIGURE S1 Total DNA (red circles) and total RNA (blue triangles) recovery efficiencies for all extracted samples over time. Recovery efficiencies were mostly between 3 and 30%.

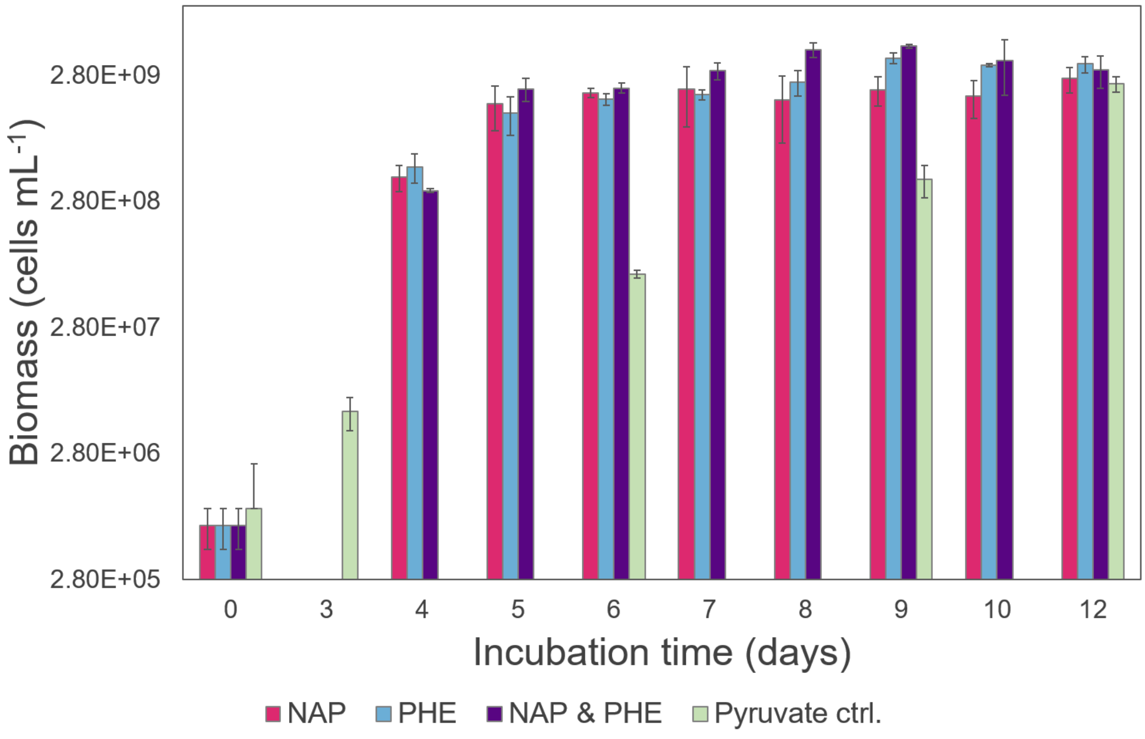

FIGURE S2 Biomass in cells mL^{-1} followed via DAPI cell counts in all experimental setups over time. Incubation conditions were naphthalene-only and phenanthrene-only (pink and blue, respectively), a mix of both PAHs (purple), and a PAH-free control, receiving pyruvate as carbon equivalent (light green). Error bars represent standard deviation between measured triplicates. At day 3, no PAH samples were measured. Pyruvate controls were sampled every third day.

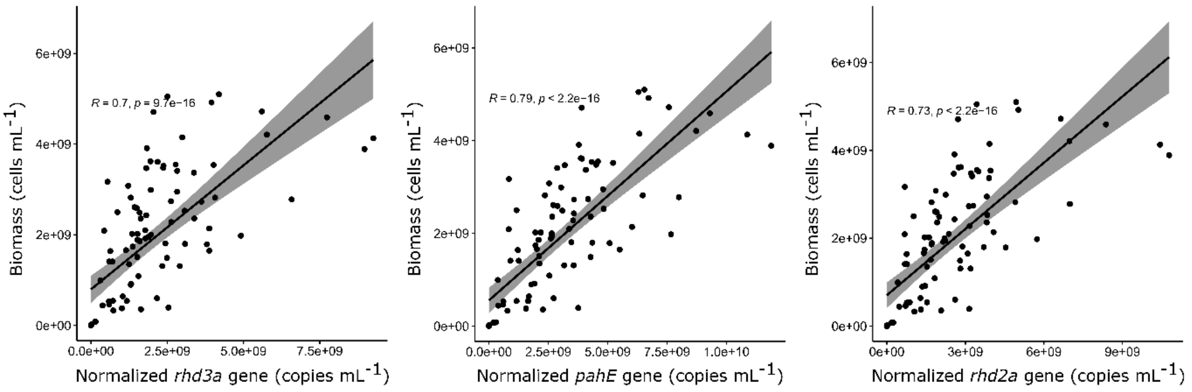

FIGURE S3 Scatter plot of biomass in cells mL^{-1} quantified by DAPI cell counts in all treatments versus the normalized gene copy number of the target genes in copies mL^{-1} (A) *rhd2α*, (B) *rhd3α* and (C) *pahE*. The linear regression line is shown with the 95% confidence interval (shaded area) including the Pearson's correlation coefficient R with the (unadjusted) p-value for each gene. Pearson’s correlation analysis with adjusted p-value is also detailed in Table S3, sheet B.

References

1. Dionisi HM, Lozada M, Marcos MS, Di Marzio WD, Loviso CL (2011). Aromatic hydrocarbon degradation genes from chronically polluted Subantarctic marine sediments. Handbook of Molecular Microbial Ecology II: Metagenomics in Different Habitats. F. J. d. Bruijn. New York, John Wiley & Sons Inc**:** 461-473.

2. Liang C, Huang Y, Wang H. 2019. *pahE*, a functional marker gene for polycyclic aromatic hydrocarbon-degrading bacteria. *Appl Environ Microbiol* 85(3).

3. Schoch CL, Ciufo S, Domrachev M, Hotton CL, Kannan S, Khovanskaya R, Leipe D, Mcveigh R, O’neill K, Robbertse B. 2020. NCBI Taxonomy: a comprehensive update on curation, resources and tools. *Database* 2020.

4. Wang W, Wang L, Shao Z. 2018. Polycyclic aromatic hydrocarbon (PAH) degradation pathways of the obligate marine PAH degrader *Cycloclasticus* sp. strain P1. *Appl Environ Microbiol* 84(21): e01261-18.
